# The Therapeutics of Laughter

**Published:** 1907-02

**Authors:** 


### EDITORIAL DEPARTMENT.

### THE THERAPEUTICS OF LAUGHTER.

The stimulating effects of laughter and jollity upon the nutritive process has been recognized from time immemorial, and it has long ago passed into a proverb, "Laugh and grow fat." This proverb, like numerous others in daily use, has, therefore, a foundation in fact; for instance, "Coming events cast their shadow before," is now recognized as thought transference—a telepathic message. How often do we find ourselves suddenly thinking of some person—the though having no connection, so far as we know, with any antecedent thought? That person has been thinking of us and we have received the message. The mind is an electrical force, as, indeed, are all vital processes, and thought transference — telepathy — is identical with wireless telegraphy. A thought is projected into space just as is a wireless message and is received by the one for whom it was intended—the mind, or receiver attuned to the proper wave lengths to receive it. Some day the world will recognize this truth, and man will be able perhaps to explain certain "psychic phenomena" which, in the existing state of knowledge, are inexplicable, and to understand why treatment of certain neuroses by galvanic or farradic electricity is curative, when all other remedies fail. The nervous system of man and of all animals is an electrical apparatus — the brain being the dynamo — for the transformation of a part of the general energy, derived from food, sunlight and air, into electricity. There are abundant grounds for this hypothesis. I need not allude to the well-known fact that there are persons whose system is so charged with electricity that sparks can be drawn from the fingers, hair, etc., by means of a suitable conductor,—a rubber comb, for instance.

The day will come, too, when when the observant and sagacious physician will make a practical application of the fact that to "laugh is to grow fat," and a fuller appreciation of the well-attested curative effects of cheerfulness. Every physician of experience knows the importance and value of a cheerful demeanor in the presence of his patient—the value of hopeful prognosis. But the influence of the mind on the glands, blood and other tissues of the body is not fully appreciated. That mental influences modify pathological conditions, even to causing the absorption of certain disease products, is well recognized. Hence, the importance of a hopeful and cheerful state of mind, both on the part of the physician and the patient and the family; for, not only does the state of the patient's mind affect the bodily condition for weal or woe, but the thoughts and other mental states of the attending physician are also felt. There is no fact in medicine better attested than that of faith healing under certain conditions; and no fact in the history of medicine better known than that certain of the English kings—notably, Charles II, the most profligate and debauched monarch that ever sat on the throne, unless I except the beast, Henry VIII—could cure scrofula by the "touch" or "laying on of hands"; and to this day scrofula is called King's Evil. How it is done, no man can explain; but the day will come when the dictum that thought is electricity, shall have been generally accepted—a rational explanation will be possible. There is no doubt in my mind that all the so-called "psychic phenomena" of clairvoyance and so-called "communications with disembodied spirits" are susceptible to some extent of explanation on the hypothesis that it all emanate from a living brain—present or absent.

The strong emotions—fear, anger, grief—are electrical storms, nerve storms, the depressing effects of which are familiar to all. They are sometimes even fatal. It is due to chemical action in the blood and nervous system, rapid chemical changes whereby morbific elements—poisons, toxines—are generated, which, before reaction comes and they can be eliminated, depress the heart and affect the mind. Who is there who reads this that can not recall an outburst of anger or fright or grief which was followed by lassitude—fatigue, prostration—and other evidences of nervous depression? A sudden shock may kill. Fear or grief has turned the hair white in an hour, and an overwhelming grief has produced long sickness and even death.

The antithesis is: the gentle emotions—the pleasurable emotions—are antidotal. Whether or not an antitoxin is liberated would be but a guess; but we all are familiar with the cheering, stimulating, tonic effects of them. If you can cheer your hypochondriac—jolly him, and make him laugh, you have used a rational remedy.

My preceptor was a fat man; and, like all fat men, he laughed and shook all over. He was always jolly, and never took a victim of the blues seriously. He had a chronic hypochrondriac for a patient. He always sent for the doctor away in the night. He lived five miles out. The doctor would cuss him a little, call for the decanter, mint and sugar, and while mixing his julep would keep up a fire of raillery, and in an hour he and the patient would be following the hounds chasing the festive fox.

It may even be said that the mental state will relieve pain. You all who are parents, can recall how mamma's kiss can cure Johnny's mashed fingers, for instance—make him forget it.

There is one notable case of the anesthetic effects of laughter on record—but not in medical archives—that is familiar even to the children. It occurs in that masterpiece of literature which has passed through perhaps many hundred thousand editions — The Melodies of *Mater Anser,* who records that

"Little Tommy Grace had a pain in his face,
So bad he couldn't learn a letter;
In came Dicky Long, singing such a funny little song,
That Tommy *laughed,*—and his face got better."

That is high authority.

Whether laughing cures by a kind of visceral massage, or whether it produces chemical changes in the blood, liberating tonic or stimulating products—perhaps alkaloidal —or *both,* I don't know. We do know, however, that "embonpoint," corpulence, at least a well-nourished body, generally co-exists with a cheerful mind, and that jollity usually characterizes a fat man. We know, too, that the stomach influences the mind, just as the state of mind influences the process of digestion. A sudden anger or fright will arrest digestion. Hence, the importance of a good digestion and of a cheerful and pleasant state of mind while and after eating. No fact is more patent than that a hungry man is cross, and it is beautiful to see him relax; "how prettily he wags his—(tongue)—whenever he is fed," or something like that.

Fat men do not commit capital crimes. They are too "good-natured" or—too lazy. They are not "fit for treason and for spoils." Shakespeare makes Julius Cæsar say, "Let me have fat men about me. Me likes not yon lean and hungry Cassius,—he thinks too much."

It is the dyspeptics, *ego,* the melancholy and morbid who make up the lean and hungry Cassiuses, the Catalines, the Guiteaus of the world. They think too much, and don't think straight.

Laughter is an electrical disturbance, not only productive of stimulating and other pleasurable and curative effects, but it is *contagious.* Who can resist the infection of the man who laughs and shakes all over? I know of no better antidote for the blue devils than The Jumping Frog or Jim Baker's Jay Bird Story. Try it.

Laugh—laugh—all you can. Make your patients laugh, and they will be better and happier, and you will be a benefactor to the human race. Selah!

